# Effects of fire on Egyptian mummies: an optical and neutron vibrational spectroscopy study

**DOI:** 10.1038/s40494-025-02261-1

**Published:** 2025-12-24

**Authors:** Maria Paula M. Marques, Victor Guida, David Gonçalves, Ana L. C. Brandão, Daniela A. H. Santos, Stewart F. Parker, Claudia Rodrigues-Carvalho, Murilo Q. R. Bastos, Luís A. E. Batista de Carvalho

**Affiliations:** 1 Department of Chemistry, Molecular Physical-Chemistry, LAQV/Requimte, University of Coimbra, Coimbra, Portugal; 2 Department of Life Sciences, University of Coimbra, Coimbra, Portugal; 3 Archaeology Graduation Program of the National Museum, Federal University of Rio de Janeiro, Rio de Janeiro, Brazil; 4 Archaeosciences Laboratory, Património Cultural I.P. (LARC/BIOPOLIS/InBIO), Lisbon, Portugal; 5 Department of Life Sciences, Centre for Functional Ecology, Laboratory of Forensic Anthropology, University of Coimbra, Coimbra, Portugal; 6 Department of Life Sciences, Research Centre for Anthropology and Health, University of Coimbra, Coimbra, Portugal; 7 ISIS Facility, STFC Rutherford Appleton Laboratory, Chilton, Didcot, UK; 8 Department of Anthropology, National Museum, Federal University of Rio de Janeiro, Rio de Janeiro, Brazil

## Abstract

Skeletal remains from mummies of the Egyptian Collection of the National Museum of Brazil, damaged by fire in 2018, were analysed by vibrational spectroscopy (infra-red, Raman and inelastic neutron scattering). Four different mummies were probed, including one from the Roman period which was one among eight worldwide. The present data delivered the burning conditions to which the mummies were subjected and allowed us to determine the extent of the heat-induced effects and the way they were impacted by the mummification process. Some of the mummies were found to have been subjected to different temperatures, depending on the location of the bone fragment in the skeleton. Several contaminants were identified; both compounds used during mummification (including salts and pigments) and construction materials from the building collapse. The results enabled us to characterise the mummified skeletal remains recovered after the fire and are expected to help establish the most suitable preservation methods.

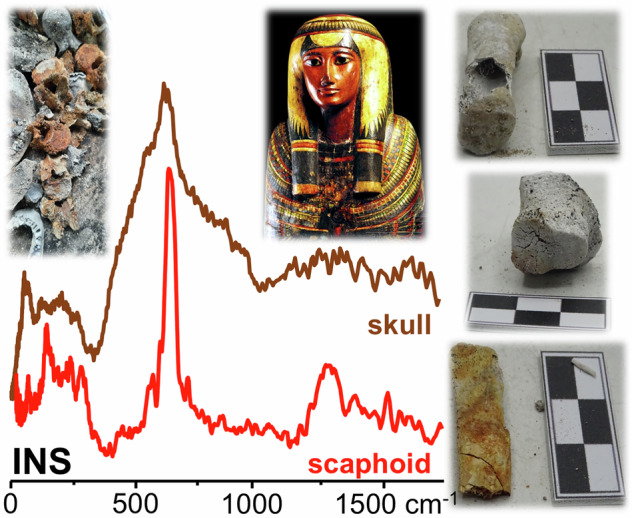

## Introduction

The Egyptian collection of the National Museum of Brazil (Rio de Janeiro) had its beginnings in 1826, when a merchant named Nicolau Fiengo sold a myriad of items to D. Pedro I (the first Brazilian Emperor). There were three mummified individuals in the collection: Hori and Harsiese, two priests from the XXI dynasty (*ca*. 1070–946 BC and *ca*.1070–767 BC, respectively) in the Third Intermediate period; and an unidentified female individual from the Roman period (*ca*. 30 BC–395 AD). This presented a distinct mummification technique only known for another eight mummified individuals worldwide^1^, which is associated with the Soter group unique to the Roman period^2–5^. Around 50 years later, in 1876, the mummy of Sha-Amun-em-su, a songstress at the temple of Karnak (Third Intermediate Period, c*ca*. 750 BC) was gifted by the Khedive Isma’il Pasha to D. Pedro II (the second Brazilian emperor) during his visit to Egypt. This was donated by him to the Museum in 1889 after the Proclamation of the Republic^6^. These Egyptian mummies have been the subject of archaeological and Egyptological studies^7^, but systematic bioanthropological analysis only began in the early 2000s^8,9^. One significant study was the tomographic analysis of the individual from the Roman period and the casket of Sha-Amun-em-su that provided relevant information on their preservation, biological profile, mummification processes and funerary goods^8,10^.

**Fig. 1:**
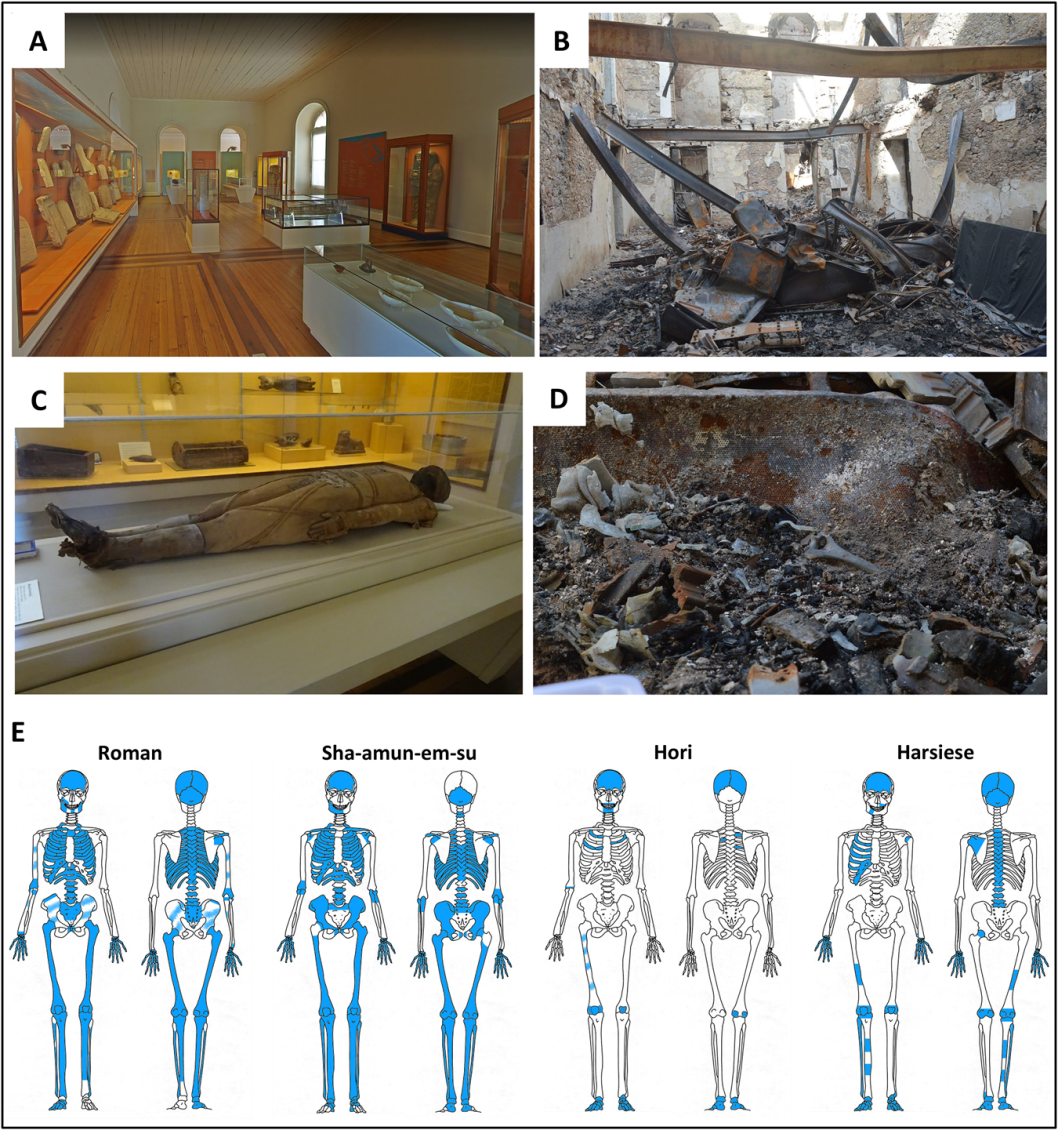
Egyptian room of the National Museum of Brazil before and after the fire in 2018. Overview of the room before (**A**) and after the fire (**B**). Mummified body of a female individual from the Roman period, before (**C**) and after (**D**) the fire showing part of a humerus. **E** Schematic representation of the skeletal remains (highly fragmented) recovered from each mummy after the fire (shown in blue)^11^. (Images: Virtual tour “Inside Brazil’s National Museum”, Google Arts&Culture (**A**); Pedro Von Seehausen (**B** and **D**); Victor Guida (**C**)).

In September 2018, a tragic fire severely affected the main palace of the Museum, compromising the natural history and anthropological collections, such as the one from ancient Egypt, that was, according to Brancaglion^6^, the largest in Latin America, comprising more than 700 artefacts. When the fire started, the four mummified individuals were on display at the Egyptian room, located on the second floor of the building, laid down inside cabinets covered by a glass lid to preserve their remains (Fig. 1 (A), (C)). Hori and Harsiese were placed next to each other at the eastern corner of the room, while the coffin of Sha-Amun-em-su was in the centre and the individual of the Roman period was at the western corner. It is noteworthy that only the Sha-Amun-em-su mummy was encased in a coffin which remained closed until it was destroyed by the fire and the collapse of the Egyptian room of the National Museum, having never been exposed before. The Hori, Harsiese and Roman mummies were wrapped in linen bandages but not in caskets. The fire was intense to the point that all the floors and the roof collapsed, throwing debris (metal furniture, wooden floor, ceiling tiles and wall construction materials) over the Egyptian remains (Fig. 1 (B), (D)). Additionally, two metal beams that supported the ceiling were bent by the heat and fell over the cabinets of Sha-Amun-em-su and of the Roman mummy. For the former, the remains rolled under the beam, while for the latter the remains were found on top of the second metal beam^11,12^. This was the scenario when the rescue team started the recovery of the Egyptian mummified remains. A significantly larger amount of bones were retrieved from the Roman and Sha-Amun-em-su mummies, with some complete bones (particularly those from the lower limbs) (Fig. 1 (D)), while for Hori and Harsiese only a few skeletal remains could be salvaged (Fig. 1 (E))^11^.

Preliminary macroscopic analysis indicated that the four mummies were affected differently, exhibiting different degrees of heat-induced transformations such as bone shrinkage and warping, colour change and thermal fractures^11^. Bone colour varied greatly (even for the same bone) within the entire colour spectrum of burned bones – creamy, brown, black, grey and white^13^. Interestingly, some fragments from Sha-Amun-em-su and the Roman mummies displayed a reddish colour, probably caused by iron contamination from the metal beams and metal furniture that fell over them^11^. Concretion, predominantly from ceiling and wall construction materials, was present in some bone fragments especially in the case of the Roman mummy, for which fragments of linen bandage used in the mummification were also found.

The destruction of these mummified corpses and their sarcophagi represents an incalculable loss for world cultural heritage and science. Currently, efforts are underway to finish the inventory of the surviving collection and to evaluate its potential for future investigations. From a bioarchaeological point of view, it is particularly important to determine the level of preservation of the mummified skeletal remains, as well as the extent of the heat induced changes which are known to occur in both the inorganic and organic bone constituents (from studies on both modern and archaeological bones)^14–19^. Some of these changes have a major impact on anthropological analyses since they may interfere with the results, especially regarding the biological profiling of the individual. Heat induced changes can provide relevant information on funerary practices^18^. Additionally, an accurate characterisation of the burned bones may offer insight into the characteristics of the fire, namely its intensity and duration, as well as on environmental conditions. Hence, a comprehensive categorisation of these mummified skeletal remains is critical to establish the most appropriate procedures for their subsequent analyses and preservation.

The present study applied the non-destructive and complementary vibrational spectroscopic techniques inelastic neutron scattering (INS, Supplementary Information)^20^, FTIR-ATR (Fourier transform infra-red in attenuated total reflection mode)^21^ and Raman spectroscopy^21^ to determine the structural and chemical variations prompted by heat (during the 2018 fire) in bones from four different mummies from the Egyptian room of the National Museum of Brazil (Table. S1 and Figure. S1, Supplementary Information). This is the first application of neutron scattering spectroscopy to the analysis of skeletal remains from mummified corpses, which is an innovative and very suitable way of tackling heat-induced transformations in these types of samples since this technique is particularly sensitive to hydrogens and is unconstrained by selection rules. The combined spectroscopic data thus obtained is expected to allow a better understanding of the burning conditions, particularly regarding: (i) skeletal preservation; (ii) taphonomical changes, specifically heat-prompted ones; (iii) biological profiles; (iv) substances used during the mummification process and the impact of this process on the heat-induced effects in bone; (v) contaminants due to the building collapse after the fire (bone contaminants having been previously detected by INS in archaeological skeletal remains)^18^. This type of knowledge is crucial for the preservation of the Museum´s Egyptian collection, which was severely affected by the fire.

## Methods

### Materials

Bones from four mummified individuals of the Egyptian Collection of the National Museum of Brazil, affected by the fire that took place in 2018, were selected for vibrational spectroscopy analysis (Table S1, Fig. S1). The selection criteria included small fragments presenting different visual taphonomic elements, representative of different anatomical regions with varying degrees of colour change. These mummified skeletal samples encompassed: (i) 6 from the Hori mummy (HO); (ii) 13 from the Harsiese mummy (HA); (iii) 10 from the mummy of the Roman period (RO); (iv) 19 from the Sha-Amun-em-su mummy (SH) (Table S1, Supplementary Information).

After gentle sanding for removal of contaminants from the outer layer, INS measurements were performed on the whole bone fragments (Fig. S1, Supplementary Information), while FTIR-ATR and Raman data were acquired for very small amounts of bone powder gathered with a scalpel from each sample. A highly crystalline sample of calcium hydroxyapatite was used as a reference—Ca_2_(PO_4_)_6_(OH)_2_ from NIST (Gaithersburg, MA, USA)^22,23^ (Ca/P = 1.67, crystallinity index = 7.91 as compared to 3.79 for poorly crystalline commercial HAp).

### Raman spectroscopy

MicroRaman spectra were recorded using a Oxford Instruments WITec (Ulm, Germany) confocal Raman microscope system alpha 300 R coupled to an ultra-high-throughput spectrometer (UHTS) 300 VIS-NIR, using a 532 nm diode-pumped solid-state laser as the exciting radiation, laser power ≤10 mW at the sample position. The measurements were acquired using a 100×/0.9 Evident MPLFLN100X objective, with 30 accumulations of 10 s per spectrum.

### Fourier transform infra-red spectroscopy

FTIR-ATR data was recorded, in the mid-IR range (400–4000 cm^−1^), in a Bruker Optics Vertex 70 FTIR spectrometer (Ettlingen, Germany) purged by CO_2_-free dry air and coupled to a Bruker Platinum ATR single reflection diamond accessory. A liquid nitrogen-cooled wide band mercury cadmium telluride (MCT) detector and a Ge on KBr substrate beamsplitter were used.

For each spectrum, 128 scans were summed at 2 cm^−1^ resolution, applying the 3-term Blackman–Harris apodization function, yielding a wavenumber accuracy better than 1 cm^−1^. The Bruker OPUS-Spectroscopy software (9.1 version)^24^ was used to correct the spectra regarding the wavelength dependence of the penetration depth of the electric field in ATR (for a mean refractive index of 1.25) and for spectral pre-processing (baseline correction and normalisation (relative to the ν_3_^as^(PO_4_) band at 1032 cm^−1^).

### Inelastic neutron scattering spectroscopy

The INS spectra were obtained at the ISIS Pulsed Neutron and Muon Source of the Science and Technology Facilities Council (STFC), Rutherford Appleton Laboratory (United Kingdom), using the time-of-flight, high-resolution broad-range spectrometers MAPS^25,26^ and TOSCA^26–28^. In MAPS, two incident energies were used (2024 and 5240 cm^−1^) in order to accurately observe all the bands from bioapatite, in both the low and high frequency ranges— namely the OH libration, its overtones and the OH stretch mode.

**Fig. 2:**
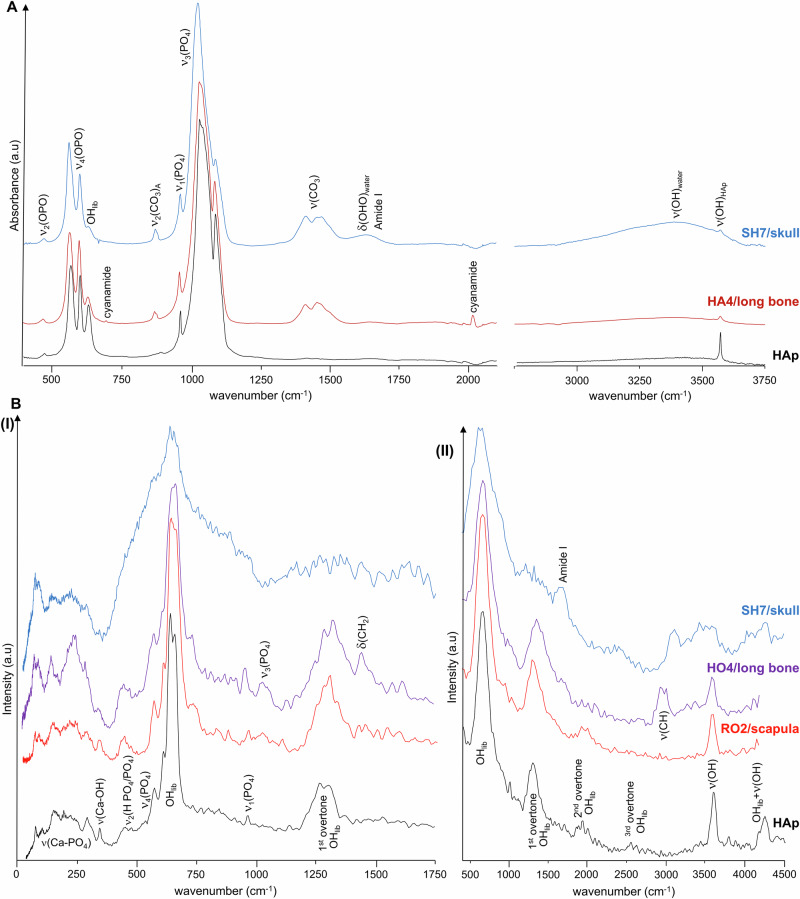
FTIR-ATR and INS spectra of mummified burned skeletal samples. **A** FTIR-ATR of Harsiese (HA) and Sha-Amun-em-su (SH); **B** INS of Roman (RO), Hori (HO) and Sha-Amun-em-su (SH), measured in TOSCA (I) and in MAPS (with 5240 cm^-1^ incident energy) (II). (The spectra of reference calcium hydroxyapatite (HAp) are shown for comparison).

The samples, whole bone fragments (1.5–4 g), were wrapped in aluminium foil and fixed onto 4 × 4 cm (TOSCA) or 5 × 5 cm (MAPS) thin-walled aluminium cans (Fig. 2 (B)). To reduce the impact of the Debye-Waller factor (the exponential term in equation (1)) on the observed spectral intensity, the samples were cooled to 5–10 K. Data were recorded in the energy range 0 to 4000 cm^−1^ (TOSCA) and 0 to 6000 cm^−1^ (MAPS), and converted to the conventional scattering law, *S*(*Q*,*ν*) vs energy transfer (in cm^−1^) using the MANTID programme (version 6.12.0)^29^.

## Results

### Bone’s vibrational profile

Application of the complementary FTIR-ATR, Raman and INS spectroscopies (optical and neutron-based) to the analysis of the burned bones of Egyptian mummies provided access to their complete vibrational profile, in a totally non-invasive way. As seen in Table 1 there are: (i) signals from the organic constituents (amide features and methyl torsions from proteins, CH_2_ deformation and stretching modes from proteins and lipids); (ii) bands ascribed to phosphate (PO_4_^3−^) and carbonate (CO_3_^2−^) groups from the bone´s inorganic framework; (iii) bioapatite´s OH translational, librational and stretching bands, clearly observed by INS but not always detected in the infra-red spectra; (iv) low frequency modes characteristic of the crystal lattice (e.g. *ν*(Ca–PO_4_) and *ν*(Ca–OH)), associated with the short-range order and hydrogen-bond network within bone´s crystalline lattice, only accessed by INS; (v) contaminants such as cyanamide, hydroxycarbonate or portlandite (Ca(OH_2_)) (debris from the building’s collapse as a result of the fire); (vi) compounds used in the mummification process.

**Table 1:** Experimental FTIR-ATR, Raman and INS wavenumbers (cm^-1^) for the burned skeletal samples from Egyptian mummies of the National Museum of Brazil

FTIR-ATR	Raman	INS	Assignment
		4255	OH_lib_ + ν(OH) (HAp)
3570	3570	3570	ν(OH) (HAp)
**3526**			**ν**_**3**_**(OH) (gypsum, CaSO**_**4**_**.2H**_**2**_**O)**
**3402**			**ν**_**1**_**(OH) (gypsum, CaSO**_**4**_**.2H**_**2**_**O)**
*ca*. 3400		*ca*. 3400	ν(OH) (water)
3285		3350	Amide A (collagen)
2850–2960		2960	ν(CH) (lipids, collagen)
		2520	3rd overtone OH_lib_ (HAp)
**2009**			**ν(NC**≡**C) (cyanamide)**
		1900	2nd overtone OH_lib_ (HAp)
**1683**			**δ(HOH) (gypsum, CaSO**_**4**_**.2H**_**2**_**O)**
1650–60	1650	1650–60	Amide I (collagen)
**1620**			**δ(HOH) (gypsum, CaSO**_**4**_**.2H**_**2**_**O)** + **δ(HOH) (H**_**2**_**O)**
	**1610**		**ν(CC)**_**G-band**_ **carbon black / ν(CO**_**3**_**)**
1550			Amide II (collagen) + ν_3_(CO_3_^2-^)_A_
1530-40			ν_3_(CO_3_)_A_ (antisymmetric stretching)
1460			ν_3_(CO_3_)_A+B_ (antisymmetric stretching)
1400–1430			ν_3_(CO_3_)_B_ (antisymmetric stretching)
	**1360**		**ν(CC)**_**D-band**_ **carbon black**
1310		1310	1^st^ overtone OH_lib_ (HAp)
**1000–1250**			**ν**_**as**_**(OSiO) (egyptian blue, CaCuSi**_**4**_**O**_**10**_**)**
**1107**	**1135**		**ν**_**3**_**(SO**_**4**_**) (gypsum, CaSO**_**4**_**.2H**_**2**_**O, antisymmetric stretching)**
1032, 1065	1076		ν_3_^as^(PO_4_) (triply degenerate) + ν_1_(CO_3_)_B_
	**1070**		**ν**_**as**_**(OSiO) (egyptian blue, CaCuSi**_**4**_**O**_**10**_**)**
	**1030**		**ν**_**2**_**(HCO**_**3**_**)**
	**1008**		**ν**_**1**_**(SO**_**4**_**) (gypsum, CaSO**_**4**_**.2H**_**2**_**O, symmetric stretching)**
962	960		ν_1_^s^(PO_4_) (nondegenerate)
915		915	ν(HCO_3_)
880			ν_2_(CO_3_)_A_
875–870			ν(HPO_4_)
870			ν_2_(CO_3_)_B_
710			ν(CO) (Ca(CO_3_))
**702**			**δ(NC** ≡ **N) (cyanamide, deformation)**
**667**	674		**ν**_**4**_**(SO**_**4**_**) (gypsum, CaSO**_**4**_**.2H**_**2**_**O, antisymmetric bending)**
**660–800**	**670**		**ν**_**s**_**(OSiO) (egyptian blue, CaCuSi**_**4**_**O**_**10**_**)**
630–660		630–650	OH_lib_ (HAp)
	**620**		**ν**_**4**_**(SO**_**4**_**) (gypsum, CaSO**_**4**_**.2H**_**2**_**O, antisymmetric bending)**
561, 575, 601	580, 593, 608	606	ν_4_(PO_4_)_bend_ (triply degenerate); ν_4_(HPO_4_)
		571	ν_2_(PO_4_)_bend_ (doubly degenerate)
	**495**		**ν**_**2**_**(SO**_**4**_**) (gypsum, CaSO**_**4**_**.2H**_**2**_**O, symmetric bending)**
		442	ν_2_(HPO_4_); ν_2_(PO_4_)
	**420–430**		**δ(SiO**_**2**_**) (egyptian blue, CaCuSi**_**4**_**O**_**10**_**)**
	**416**		**ν**_**2**_**(SO**_**4**_**) (gypsum, CaSO**_**4**_**.2H**_**2**_**O, symmetric bending)**
	**335**		**ν(HgS) (cinnabar, HgS)**
		290–330	ν_3_(Ca–OH); OH_trans_ (HAp)
	**260**		**ν(HgS) (cinnabar, HgS)**
		250	τ(CH_3_) (collagen, torsion)
		190–230	(Ca–PO_4_)_lattice_
	**179**		**H**_**2**_**O lattice mode (gypsum, CaSO**_**4**_**.2H**_**2**_**O)**
		130–150	OH_trans_ (water) + (Ca–PO_4_)_lattice_
	**137**		**ν(PbO) (litharge, tetragonal PbO)**
		79	ν(Ca–PO_4_)_lattice_

Contributions from mummification substances and contaminants (such as mummification compounds and construction materials) are represented in bold case.

The band from ν_3_^as^(PO_4_) dominates the infra-red spectra, at 1032 cm^−1^ (Fig. 2 (A)), while the most prominent INS features are the OH librational and stretching modes from bone´s bioapatite (at 630-650 and 3570 cm^-1^, respectively), the latter is particularly well observed in the spectra measured in MAPS (at 5240 cm^-1^ incident energy) (Fig. 2 (B)). Regarding the INS measurements, the concomitant use of MAPS and TOSCA spectrometers enabled access, with very high sensitivity, to both the low frequency spectral region (<1000 cm^−1^ in TOSCA) and the high wavenumber range (1000–4000 cm^−1^ in MAPS). These data revealed different burning conditions for the four mummies under study (as expected since they were in different sections of the gallery), mostly evidenced by the low frequency modes (best observed in TOSCA) which have been shown to be good spectroscopic markers of heat-induced changes within bone´s crystalline framework^15,17,30,31^. HAp’s librational band, centred at *ca*. 640 cm^−1^; ν_2_(PO_4_) and ν_2_(HPO_4_) at *ca*. 470 cm^-1^; ν_3_(Ca-OH) (at *ca*. 330 cm^−1^), which was previously found to be observed only above 700 °C (upon OH for phosphate substitution within bioapatite)^31^, OH translations and Ca–PO_4_ lattice modes (70–120 cm^-1^) (Fig. 2 (B-I)).

### Characterisation of contaminants

The sophisticated preservation procedures that were used by the Egyptians (from *ca*. 4500 BC to hundreds of years AD)^32^ for transforming a corpse into a lasting mummy (a process that could take up to 70 days) involved removing the brain (excerebration, without filling the cavity) and the internal organs (evisceration), applying different preservation and stuffing materials and finally linen bandages to wrap the body. This process encompassed anthropogenic desiccation and the use of antioxidants, antibacterials, antifungals, barrier materials, fixatives and fragrances to preserve the tissue and avoid unpleasant smells^33–37^. These substances comprised conifer oils, pistacia and elemi resins, animal fats, dammar (triterpene), beeswax (fatty acid esters and alcohols), arabic gum (polysaccharide) and petroleum bitumen (hydrocarbon). Of these, the lipidic compounds were not detected in the spectra of the burned mummified bones currently studied, since they probably acted as fuels which fed the fire locally and were finally destroyed by it—hence the absence of the characteristic δ(CH_2_) (1400–1450 cm^−1^), ν(CH_2_) (2850–3000 cm^−1^) and ν(C=O)_ester_ (*ca.* 1720 cm^−1^) signals, the latter having been previously detected by FTIR in unburned skin remains of Egyptian mummies^38,39^. In turn, the INS bands observed for some samples in the high wavenumber region of the spectra, between 2850 and 3000 cm^−1^, are assigned to CH_2_ symmetric and antisymmetric stretching modes mostly arising from the protein constituents of bone when subjected to low-to-moderate temperatures (<500–600 °C, e.g. detected in the HO4 sample, Fig. 2 (B-II)). The signal at *ca*. 1650 cm^−1^ observed for some of the mummified specimens by FTIR (Figure. SH7, Fig. 2 (A)) is ascribed to the Amide I vibrational mode from proteins (ν(C=O)), either from the bone itself (collagen, only destroyed at temperatures above 500–600 °C^17,31,40^ or from the balms added during mummification (in some samples, namely SH7, this band is broadened by the presence of δ(HOH) signal from water).

Natron (salt of Amun) is another mummification substance, essential for the desiccation of the cadaver prior to its treatment with oils and resins, to prevent fungal and bacterial attack and subsequent tissue degradation. It is a mixture of hydrated sodium carbonate (Na_2_CO_3_.10H_2_O) and sodium bicarbonate (NaHCO_3_), with small amounts of NaCl and Na_2_SO_4_, found in the crystalline form on the banks of several lakes in Egypt (e.g., Wadi Natrun, in the Nile delta). It is reported to yield medium to strong Raman signals at *ca*. 1045/1076 cm^−1^ (from HCO_3_/CO_3_ stretching modes)^37,41,42^. Although the presence of this compound in the mummified skeletal samples under study is highly probable, its characteristic bands could not be detected (probably hidden by those from bone´s phosphates and carbonates, respectively at 1030–1092 and 1400–1460 cm^−1^) (Table 1). Additionally, the thermal decomposition of natron within the burned mummified bones should be taken into account – it proceeds through several dehydration steps at increasing temperatures, yielding sodium carbonate monohydrate (thermonatrite, Na₂CO₃·H₂O) and anhydrous sodium carbonate (natrite, Na₂CO₃), the latter decomposing to sodium oxide and carbon dioxide above 500 °C.

**Fig. 3:**
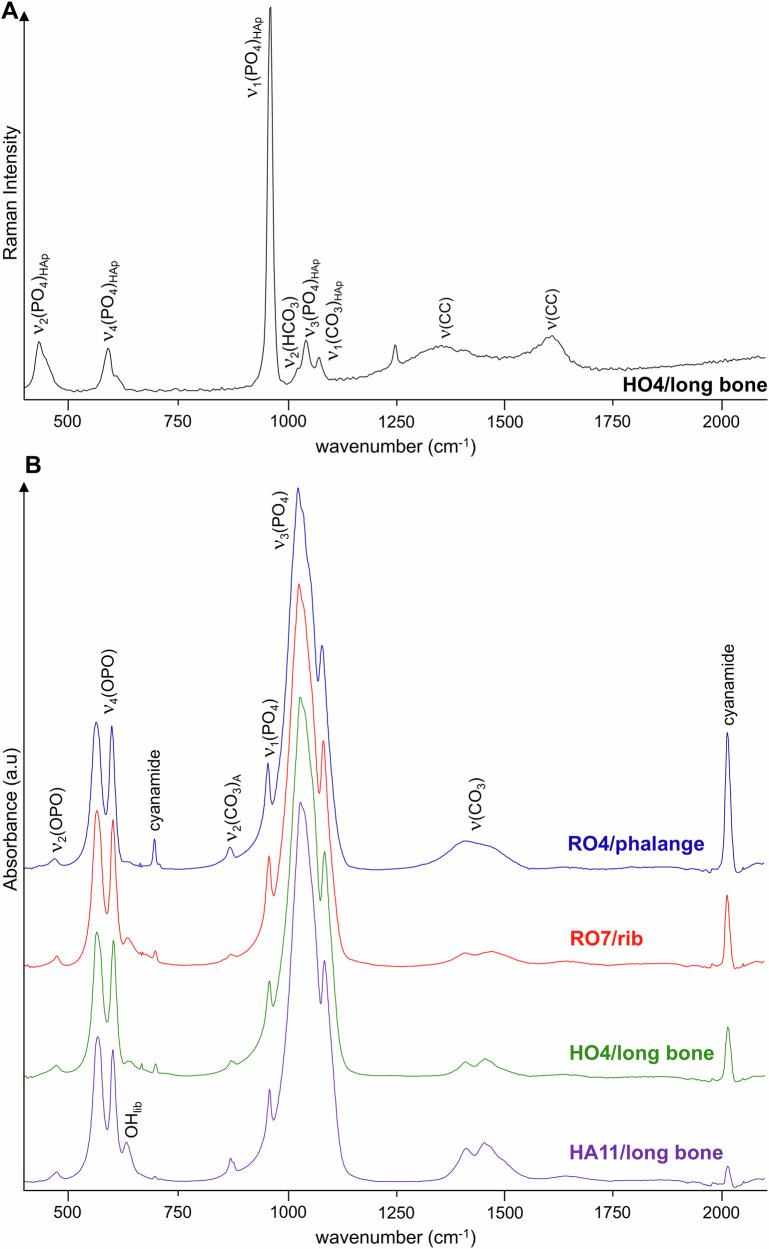
Raman and FTIR-ATR spectra of mummified skeletal samples. **A** Raman spectrum of a mummified burned fragment of a long bone from Hori (HO4), showing bands from carbon black. **B** FTIR-ATR spectra of mummified burned skeletal samples from Roman (RO), Hori (HO) and Harsiese (HA), showing the presence of cyanamide. (HAp – hydroxyapatite).

**Fig. 4:**
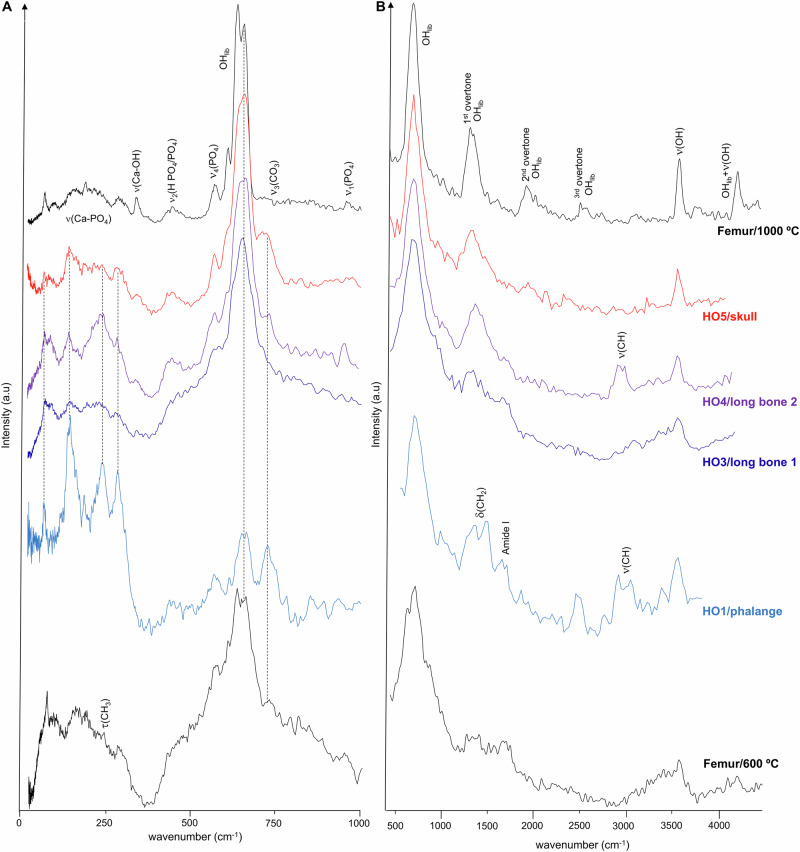
INS spectra of mummified burned skeletal samples from Hori (HO). **A** Measured in TOSCA; **B** measured in MAPS, with 5240 cm^−1^ incident energy. (The spectra of modern human femur burned aerobically at controlled temperatures are also shown for comparison^31^).

The very intense narrow feature centred at 960 cm^−1^ (Fig. 3) is ascribed to the phosphate ν_1_(PO_4_) mode. In some of the mummified samples, namely for Harsiese (HA11/long bone), Sha-Amun-em-su (SH17/rib), Roman (RO7/rib) and Hori (HO4/long bone), a typical Raman ν_2_(HCO_3_) band was detected at 1030 cm^−1^ (Fig. 3 (A)). Moreover, the Raman profiles of these samples evidenced signals at 1360 and 1610 cm^−1^, which are ascribed to D and G-bands from amorphous carbon, respectively^43^ (Table 1). In addition, the INS spectra of the HO1 and RO1 samples evidence a loss of hydroxyls from bone’s inorganic framework, in agreement with the FTIR results—for several samples no OH libration signal (660 cm^−1^) is detected (Fig. 3 (B)) and the ν(OH) band (3570 cm^−1^) has a low intensity (Fig. 4 (B)). This may be associated with bone degradation processes, mostly due to the extensive use of natron in the mummification which was recently reported to cause erosion in bone (detected through an increase in porosity)^37^, that might have left the mummified samples currently analysed more prone to the heating effects during the fire.

Additional vibrational features arising from cyanamide (CN_2_H_2_) are observed by FTIR in some bone fragments from the Roman, Hori and Harsiese mummies—at 702 cm^−1^ (δ(NC≡N)) and 2009 cm^−1^ (ν(NC≡N)) (Table 1, Fig. 3 (B)). This compound was reported to be formed during burning events under reduced oxygen availability (anaerobic or quasi-anaerobic conditions), from NH_3_ released from bone’s organic constituents (namely collagen), CN_2_^2-^ being incorporated into the bone matrix to yield Ca_10_(PO_4_)_6_CN_2_^17,19,44^. During the fire at the Egyptian room of the museum, the building collapse might have led to the mummies being buried, which could then have been partially deprived of oxygen during burning. This contaminant may also be due to the presence of ammonia in the remains, namely from NH_4_Cl and balms used during the mummification process, which was transformed into cyanamide upon heating. It is noteworthy that no cyanamide was detected in any of the samples from Sha-Amun-em-su, which was the only mummy inside a coffin.

**Fig. 5:**
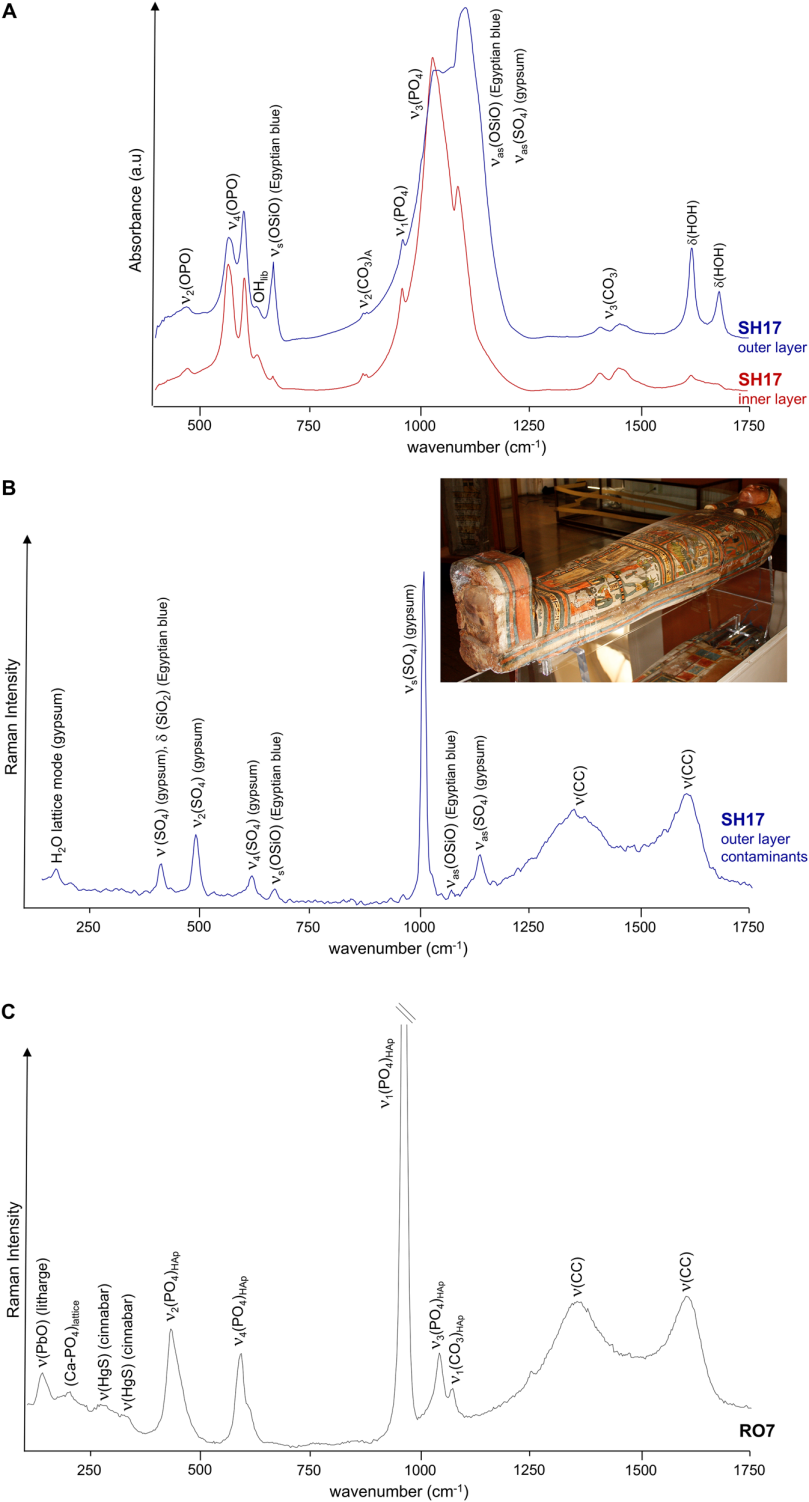
FTIR-ATR and Raman spectra of mummified burned skeletal fragments. Sha-Amun-em-su (rib, SH17) samples collected from the outer and inner layers – **A** FTIR-ATR, and from the outer layer contaminants showing the presence of gypsum – **B** Raman; Roman mummy (rib, RO7) evidencing the presence of litharge and traces of cinnabar – Raman (**C**). The inset shows Sha-Amun-em-su´s coffin, before the fire.

One of the rib fragments from Sha-Amun-em-su (SH17) yielded interesting data (Fig. 5): FTIR and Raman spectra obtained from the outer layer of the sample evidenced a high amount of contaminants, among which high amounts of gypsum (CaSO_4_.2H_2_O) mainly revealed by its typical infra-red bands at 1620 and 1683 cm^−1^ ascribed to deformation bands of crystal water—from two crystallographically non-equivalent water molecules (Table 1, Fig. 5 (A))^16,41,45^. Gypsum is a constituent of Portland cement (the most common type of cement in use since the XIX century as a basic ingredient of concrete, mortar and stucco), and was also used in ancient civilisations mixed with pigments or dyes, and a gypsum layer was often applied prior to painting. Hence, its detection in these mummified skeletal samples is suggested to be predominantly due to the presence of pigments, both from the mummy itself (since the mummies were sometimes painted in red (men) or yellow (women)^46^) and from the painted coffin.

Additionally, pigments were detected in the external sample of the SH17 rib (and not in the inner layer) (Fig. 5 (A) *vs* (B)): Egyptian blue (cuprorivaite, CaCuSi_4_O_10_), through the Raman bands at 420 (δ(SiO_2_)), 670 (ν_s_(O-Si-O)) and 1070 cm^−1^ (ν_as_(O-Si-O)), and possibly also by the FTIR features at 670 cm^−1^ (ν_s_(O-Si-O)) and *ca*. 1100 cm^−1^ (ν_as_(O-Si-O))^47,48^ the former being detected in the outer layer, but not in the inner layer, of sample SH17, while the latter may be overruled by the intense and narrow gypsum ν_s_(SO_4_) signal at 1008 cm^−1^ ^47,49–53^; carbon black, contributing to the Raman bands centred at *ca*. 1360 and 1610 cm^−1^ ^43,49,54^. Regarding thermal stability, gypsum undergoes dehydration at high temperatures to form anhydrous CaSO_4_, while Egyptian blue is stable at temperatures well above 1000 °C. Calcium oxalate was reported to be a product of long-term degradation of mummified skin, and it can also be present as salt efflorescences on wall paintings and marbles^38,53^. However, its presence intermingled with the mummified skeletal samples was not currently detected. Interestingly, the spectra measured for the inner layer of SH17 hardly showed any signals from contaminants (Fig. 5 (A)), as neither pigments nor compounds from the building collapse would be expected to be present in the inner part of the bones. Detection of contaminants from construction materials (Fig. 5 (B)) allowed us to infer on specific environmental settings related to the fire at the Egyptian Room of the National Museum. Before the fire, Sha-Amun-em-su was the only mummy from the Egyptian room that was inside a coffin, the other three being simply wrapped in linen bandages.

Other types of pigments known to have been used in Ancient Egypt were detected for some of the samples, namely for the Roman mummy: red-to-yellow litharge (PbO), yielding a characteristic ν(PbO) Raman band at 137 cm^−1^ ^23^ observed for all specimens from this mummy except for a scapula fragment (RO2); and traces of cinnabar (HgS), giving rise to weak ν(HgS) Raman signals at 260 and *ca*. 335 cm^−1^ (Table 1, Fig. 5 (C)). Cinnabar, in particular, was only applied in the decoration of Egyptian artefacts during and after the Greco-Roman period (Ptolemaic period, 332 BC–395 AD)^47^, which justifies the fact of having been detected only in the samples from the Roman mummy. The corresponding Raman bands are much less intense than for the pure pigment^47^, which is expected if only traces are to be found in these skeletal burned remains, highly contaminated with construction materials. At increasing temperatures cinnabar can decompose to SO_2_ releasing mercury vapour.

For some of the mummies probed distinct heating temperatures were identified within the same skeleton (from both the INS and FTIR results), namely for the Hori priest: a phalange (HO1) and a long bone sample (HO4, femur or tibia) yielded spectra consistent with a low-to-moderate heating temperature (≤400 °C), protein´s Amide I band (at 1650 cm^-1^) and ν(CH) from lipids and collagen (at *ca*. 3000 cm^-1^) still being observed by FTIR and Raman spectroscopy (as compared to a modern femur specimen heated at 600 °C)^21^ (Fig. 4), while another long bone fragment (HO3) evidenced a moderate heating (500-700 °C). A fragment from skull from the same mummy (HO5), in turn, revealed a severe burning ( ≥ 900 °C), its spectral profile being identical to that measured for a modern femur sample burned at 1000 °C^31^, showing no traces of protein or lipids and a narrow OH librational band (at 660 cm^-1^, best detected in TOSCA) (Fig. 4).

**Fig. 6:**
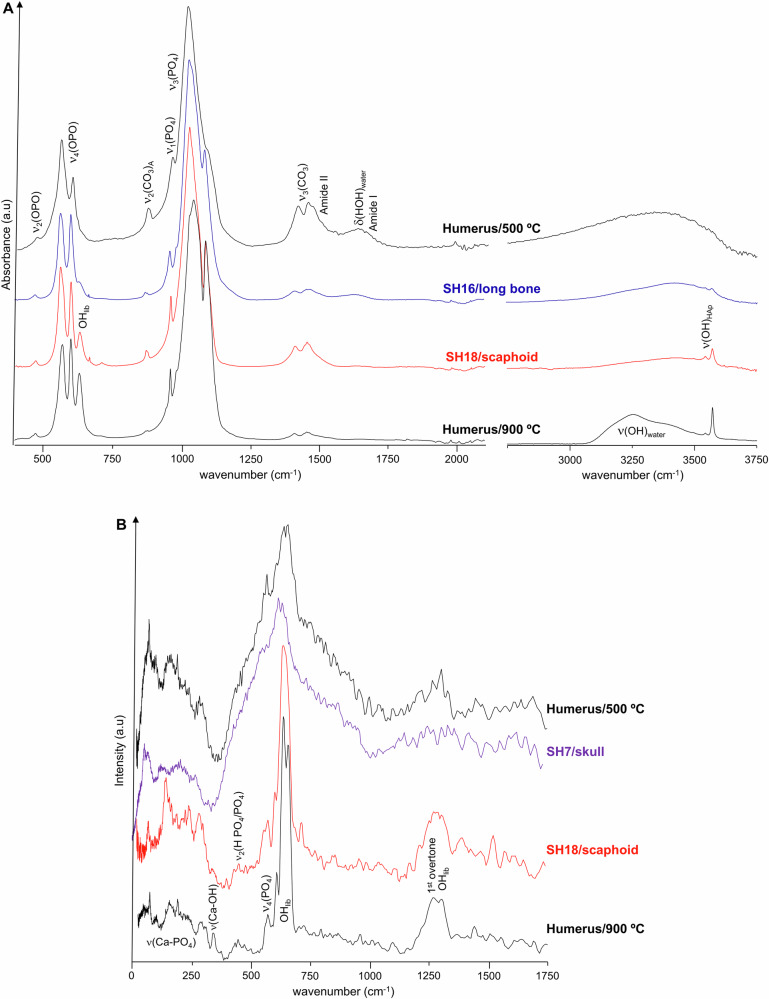
FTIR-ATR and INS spectra of mummified burned skeletal samples from Sha-Amun-em-su (SH). **A** FTIR; **B** INS (measured in TOSCA). (The spectra of modern human humerus burned aerobically at controlled temperatures are also shown for comparison^31^).

Similarly, for the Sha-Amun-em-su mummy the heating effect was sensed differently by the scaphoid bone (SH18) as compared to other bone samples under analysis, such as the skull (SH7) and a long bone (SH16) (Fig. 6). The FTIR profiles depict a distinctive OH librational band for the scaphoid, as opposed to the long bone (Fig. 6 (A)), in agreement with a high burning temperature (*ca*. 900 °C, as compared to the reference spectrum of human humerus heated at this temperature). In addition, a very significant broadening was observed for the INS (OH)_lib_ feature for the skull (SH7) *vs* the scaphoid (SH18) (Fig. 6 (B)), consistent with a much lower heating temperature (as shown by comparison with the spectra of modern humerus burned at 900 and 500 °C). The Roman mummy also evidenced distinct burning temperatures, although not as striking as for Hori and Sha-Amun-em-su. For Harsiese, in turn, the spectral data revealed quite uniform profiles for all samples except HA3, consistent with high heating temperatures. These different impacts of the fire even for the same mummified skeleton had previously been observed macroscopically (by analysis of the distinct bone colours^11^, Fig. S1 (A), Supplementary Information), and may be due to several factors: (i) the direction of the fire and main heat source in the vicinity of the mummy; (ii) since the mummies were placed on a metal surface (covered by glass), the heat may have been transmited differently to the distinct parts of the corpse, reaching higher temperatures nearer to the metal structure; (iii) upon the collapse of the building the flammable debris may have fallen on different sites of the same mummy, generating hotspots. Additionally, it is possible that some of the construction materials acted as a shield against the fire – this probably happened for Sha-Amun-em-su, for which a metal beam that fell from the ceiling covered the lower limbs, larger bone fragments than for the other mummies having been found; (iv) a different protection of the distinct parts of the skeleton due to the mummification process, namely different bandaging in the different sections of the corpse which may result in different buffering effects against heating.

## Discussion

The main goal of this study was to identify spectral variations associated with chemical and structural changes in human bone samples from Egyptian mummies, prompted by the fire that took place in the National Museum of Brazil in 2018. This is an innovative study, reporting the first application of vibrational spectroscopy, including neutron scattering techniques, to the analysis of skeletal samples from mummified bodies (in a non-invasive/non-destructive way).

The combined INS, infra-red and Raman spectroscopy results presented here have enabled a thorough interpretation of the vibrational data gathered for the burned mummified skeletal samples, never probed before by these techniques. Inelastic neutron scattering, in particular, allowed to detect all the modes associated to bone´s hydroxyl groups (translational, librational and stretching), not all accessed by infra-red, as well as the low frequency vibrations characteristic of the crystal lattice. Additionally, the concomitant INS measurements at MAPS and TOSCA delivered a consistent set of data which was key for achieving an accurate assignment the vibrational modes and therefore an accurate identification of spectral biomarkers of heat-prompted changes in bone as well as of the impact of the mummification process on these variations.

Some spectral features could be identified as reliable biomarkers of heat-prompted lesions in human mummified bone, as well as of the presence of contaminants (both from the mummification process and the construction materials) (Table 2). These allowed us to attain a rough estimate of the temperatures to which the samples were subjected. These data provide reliable clues for a precise characterisation of the burning and environmental conditions during the fire at the National Museum.

**Table 2:** Main results regarding heat-induced changes and presence of contaminants detected (by FTIR-ATR, Raman spectroscopy or INS) in the burned mummified skeletal samples from Egyptian mummies of the National Museum of Brazil

Sample	Tentative burning temperature	Detected compound	Vibrational biomarker (cm^−1^)	Detected by
		protein	ν(CH) – 2850–3000	INS
HA3, HO1, HO3, HO4, SH7, SH8, RO1, RO6	<400–500 °C	protein	Amide I – 1650	FTIR
		protein	τ(CH_3_) – 250	INS
SH17 (outer layer)	800–900 °C	gypsum	δ(HOH) – 1620, 1683 ν_4_(SO_4_) – 620 ν_1_(SO_4_) –1008 ν_3_(SO_4_) –1130	FTIR Raman
			H_2_O lattice mode - 179 ν(SO_4_) – 1107	Raman
		hydroxyapatite OH´s	OH libration – 660	INS, FTIR
		egyptian blue	δ(SiO_2_) – 420-430 ν_s_(OSiO) – 670 ν_as_(OSiO) – 1070	Raman FTIR, Raman Raman
		carbon black	ν(CC) – 1360, 1580	Raman
HA7, HA9, HA10, HO5, SH1, SH12, SH14, SH18, RO2, RO5, RO7, RO9	>900 °C	hydroxyapatite OH´s cinnabar (traces)	OH libration – 660 ν(OH) – 3570 ν(HgS) – 260, 335	INS, FTIR
HA11, HO4, SH17, RO7		CO_3_^2-^, HCO_3_^-^	ν(CO_3_) – 1360, 1610	Raman
RO7		egyptian blue	δ(SiO_2_) – 420-430 ν_s_(OSiO) – 670 ν_as_(OSiO) – 1070	Raman FTIR, Raman Raman
HA5, HA8, HA11, HA12, HO4, RO4, RO7, RO8, RO9		cyanamide	δ(NC≡N) – 702 ν(C≡N) – 2009	FTIR

Tentative temperatures to which the samples were subjected are also shown.

## Supplementary information

Supplementary Information

## Data Availability

The FTIR and Raman data will be available from Molecular Physical-Chemistry—LAQV/Requimte, University of Coimbra, Portugal. The INS dataset supporting this article is available from the Science and Technology Facilities data repository, UK (eData) (doi: 10.5286/ISIS.E.RB2410051, doi: 10.5286/ISIS.E.RB2410053).
