# Supplementary material for: Effects of fire on Egyptian mummies: an optical and neutron vibrational spectroscopy study
Source: NPJ Herit Sci. 2025 Dec 24;13(1):682. doi: 10.1038/s40494-025-02261-1 (PMC12738286; doi:10.1038/s40494-025-02261-1)
Supplement: Supplementary file 1 — Supplementary Information [file 40494_2025_2261_MOESM1_ESM.pdf]

Supplementary Information

Effects of Fire on Egyptian Mummies: An Optical and Neutron Vibrational Spectroscopy Study

Maria Paula M. Marques,[1,2] *Victor Guida,*[3] *David Gonçalves,*[4,5,6] *Ana L.C. Brandão,*[1,2] *Daniela A.H. Santos,*[1] *Stewart F. Parker,*[7] *Claudia Rodrigues-Carvalho,*[8] *Murilo Q.R. Bastos,*[8] *Luís A.E. Batista de Carvalho*[1*]

*Corresponding author. Email: labc@ci.uc.pt

This file includes:

Tab. S1 – Mummified skeletal samples from Egyptian mummies of the National Museum of Brazil (subjected to fire) analysed in this study.

<ins>Composition and Spectroscopic Signature of Bone</ins>

Fig. S1 – (A) Some of the mummified skeletal samples from the 2018 fire analysed in this study from the Hori (HO) and Roman (RO) mummies. (B) Bone fragments wrapped in aluminium foil and fixed onto flat aluminium sample holders for INS measurements.

Fig. S2 – FTIR-ATR (A), Raman (B) and INS (C) spectra of the mummified skeletal samples from Egyptian mummies of the National Museum of Brazil (subjected to fire) analysed in this study (listed in Table S1).

Table S1. Mummified skeletal samples from Egyptian mummies of the National Museum of Brazil (subjected to fire) analysed in this study.

Skeletal fragment	Designation	Skeletal fragment	Designation
Hori		Roman	
phalange	HO1	rib	RO1
phalange	HO2	scapula (shoulder blade)	RO2
long bone	HO3	phalange	RO4
long bone	HO4	vertebra	RO5
skull	HO5	vertebra	RO6
pelvis *or* epiphysis of a long bone	HO6	rib	RO7
		foot	RO8
		hand	RO9
Harsiese		Sha-Amun-em-su	
long bone	HA1	navicular bone (tarsus)	SH1
rib	HA2	phalange	SH2
rib	HA3	navicular bone (tarsus)	SH3
long bone	HA4	phalange	SH4
long bone	HA5	phalange	SH5
long bone	HA6	rib	SH6
metatarsus	HA7	skull	SH7
long bone	HA8	skull	SH8
phalange	HA9	skull	SH9
radius	HA10	rib	SH10
long bone	HA11	phalange	SH11
skull	HA12	metatarsus	SH12
long bone	HA14	phalange	SH13
		cuboid bone (tarsus)	SH14
		rib	SH15
		long bone	SH16
		rib	SH17
		scaphoid bone (carpus)	SH18
		phalange	SH19
		rib	SH20

Composition and spectroscopic signatures of bone

Bone is a heterogeneous material comprising (apart from water) proteins (mostly collagen I) and lipids, within an inorganic matrix of hydroxyapatite ($Ca_{10}(PO_4)_6OH_x$, HAp). Of the bone's total mass, 60% is ascribed to the inorganic phase (increasing to 70% in dry bone), *ca.* 25% to the organic components and 9.7% to water. In HAp the hydroxyl and phosphate groups are partly substituted by carbonate (A- and B-type, respectively), yielding $Ca_{10}(PO_4)_{6-x}(OH)_{2-y}(CO_3^{2-})_{x+y}$ (bioapatite).[1,2] This framework may also contain other ions such as sodium (Na^+), magnesium (Mg^{2+}), strontium (Sr^{2+}), potassium (K^+), fluorine (F^-) or chlorine (Cl^-).[3,4] The specific composition of bone, as well as its crystalline structure, depends on several factors, namely diet, metabolism, pathologies, age at death, post mortem period, burning events and environmental conditions. Due to their high sensitivity and specificity coupled to a virtually non-destructive nature, vibrational spectroscopy techniques – Raman, Fourier transform infrared (FTIR) and inelastic neutron scattering (INS) spectroscopies – have been shown to be very effective for analysing bone samples, either forensic or archaeological, burned and unburned.[5-18] These analytical methods deliver accurate information on the chemical and

structural features of the skeletal specimens, without the need for sample preparation, providing reliable clues for a precise characterisation of the conditions that have shaped the bones (e.g. heating events, environmental contamination or pathological disorders). Several studies have reported the application of Raman and FTIR to the analysis of mummified remains (*e.g.* to determine their state of conservation and identify embalming substances), namely bone, hair, skin and linen bandages from Peruvian and Egyptian mummies.[19-27]

Inelastic neutron scattering (INS) is a non-optical vibrational spectroscopy technique, complementary to Raman and FTIR, particularly suitable for probing hydrogenous materials such as bone. In INS, the intensity of each vibrational transition is expressed, for a given atom, by the dynamic structure factor

$$\mathbf{S_i^*(Q, \nu_k)} = \frac{(\mathbf{Q^2 u_i^2})\sigma}{\mathbf{3}} \mathbf{exp}\left(-\frac{\mathbf{Q^2 \alpha_i^2}}{\mathbf{3}}\right) \quad (1)$$

Q ($Å^{-1}$) representing the momentum transferred to the sample, $\nu_\mathbf{k}$ the energy of a vibrational mode, u_i (Å) the displacement vector of atom *i* in mode *k*, σ the neutron scattering cross section of the atom and α_i (Å) the mass-weighted sum of the displacements of the atom in all vibrational modes. There are no selection rules for INS (as opposed to FTIR and Raman), which allow us to observe all the fundamental vibrations, overtones and combination bands for the samples under analysis. Previous INS studies of human skeletal remains have provided very accurate information on bone´s composition, as well as on heat-induced chemical substitutions within the bone matrix (*e.g.* at the hydroxyl sites) and on changes in carbonate and water content.[5,6,10,11,13,28-32]

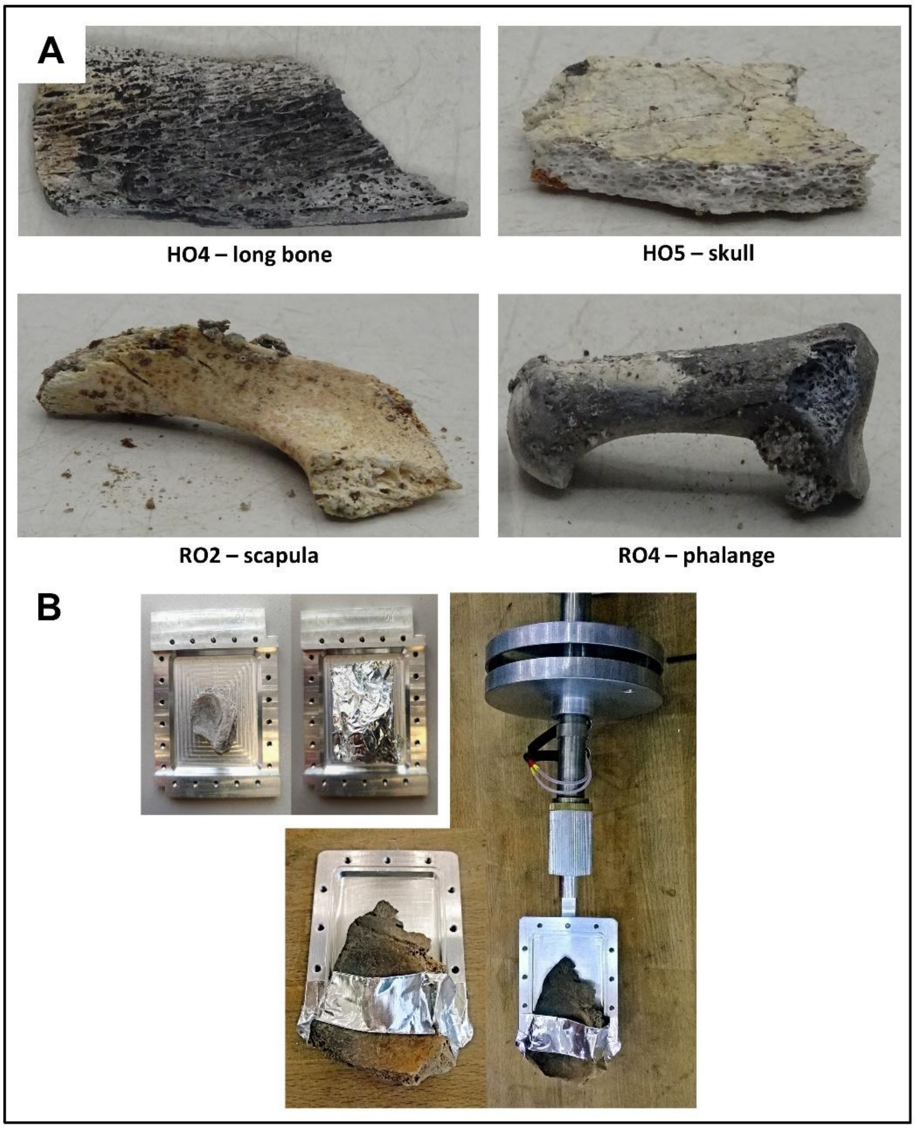

Figure S1. (A) Some of the mummified skeletal samples from the 2018 fire analysed in this study from the Hori (HO) and Roman (RO) mummies. (B) Bone fragments wrapped in aluminium foil and fixed onto flat aluminium sample holders for INS measurements.

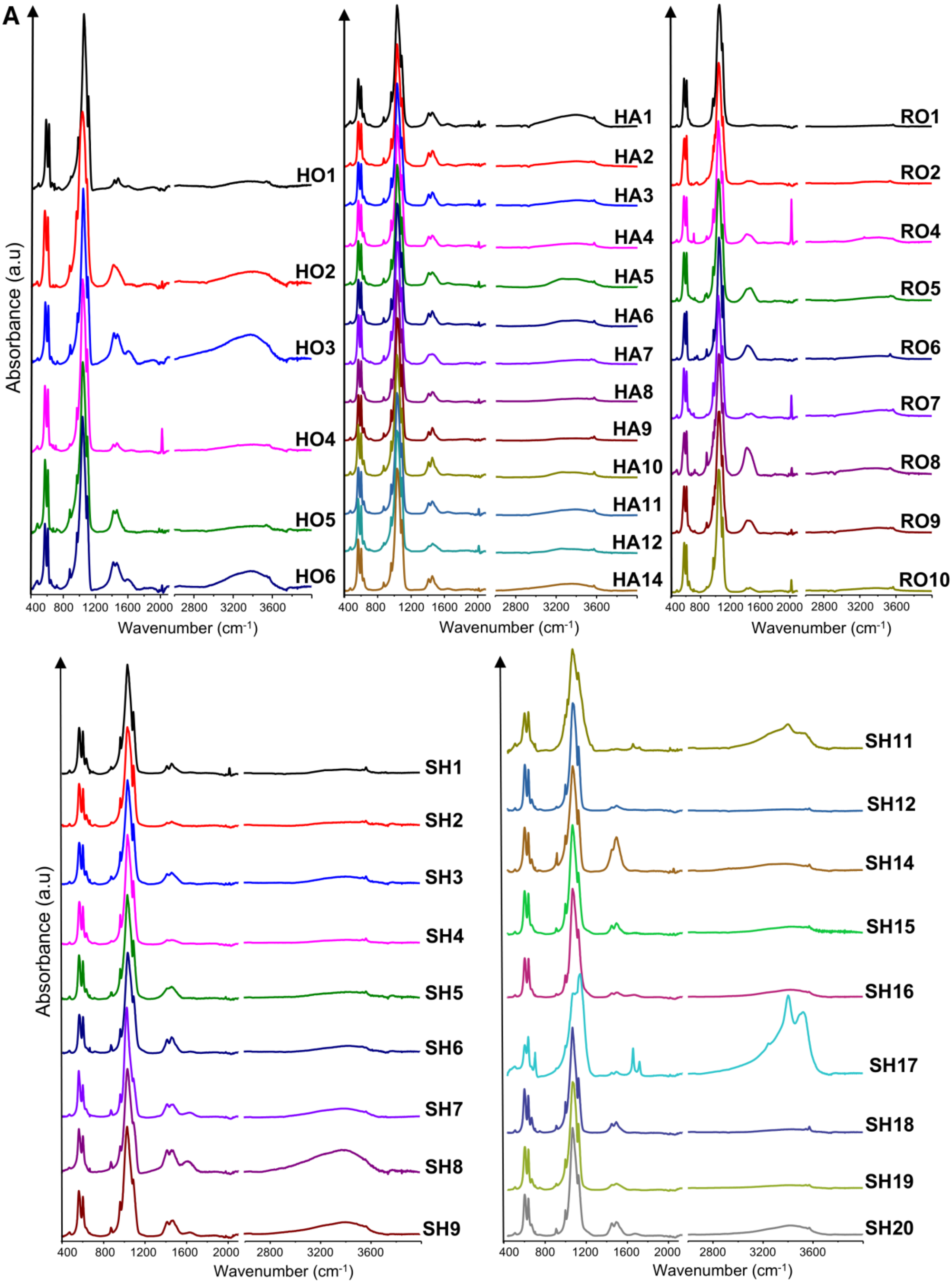
A
Absorbance (a.u)
HO1
HO2
HO3
HO4
HO5
HO6
400 800 1200 1600 2000 2800 3200 3600
Wavenumber (cm-1)
HA1
HA2
HA3
HA4
HA5
HA6
HA7
HA8
HA9
HA10
HA11
HA12
HA14
RO1
RO2
RO4
RO5
RO6
RO7
RO8
RO9
RO10
SH1
SH2
SH3
SH4
SH5
SH6
SH7
SH8
SH9
SH11
SH12
SH14
SH15
SH16
SH17
SH18
SH19
SH20

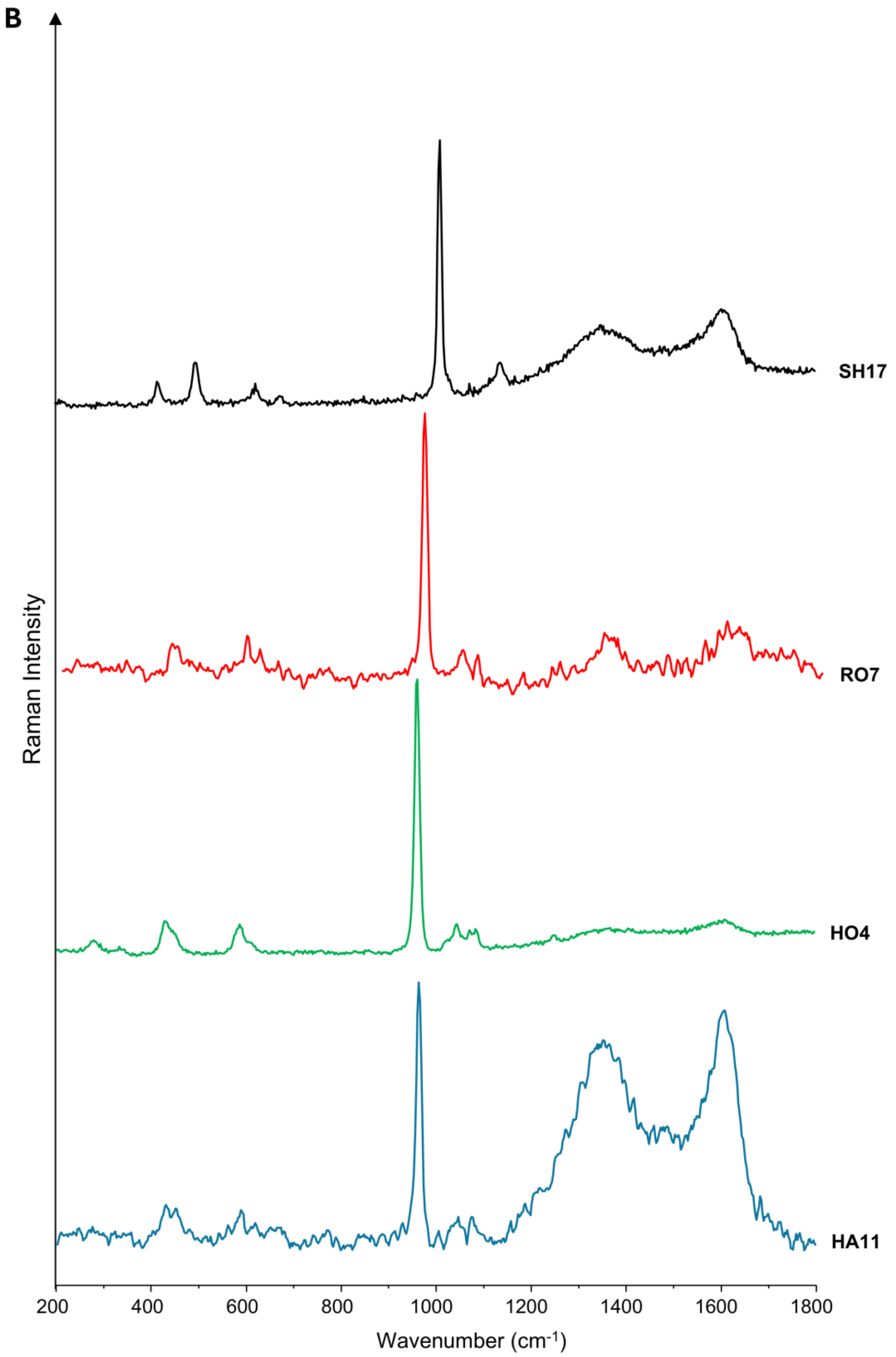
B
Raman Intensity
SH17
RO7
HO4
HA11
200
400
600
800
1000
1200
1400
1600
1800
Wavenumber (cm-1)

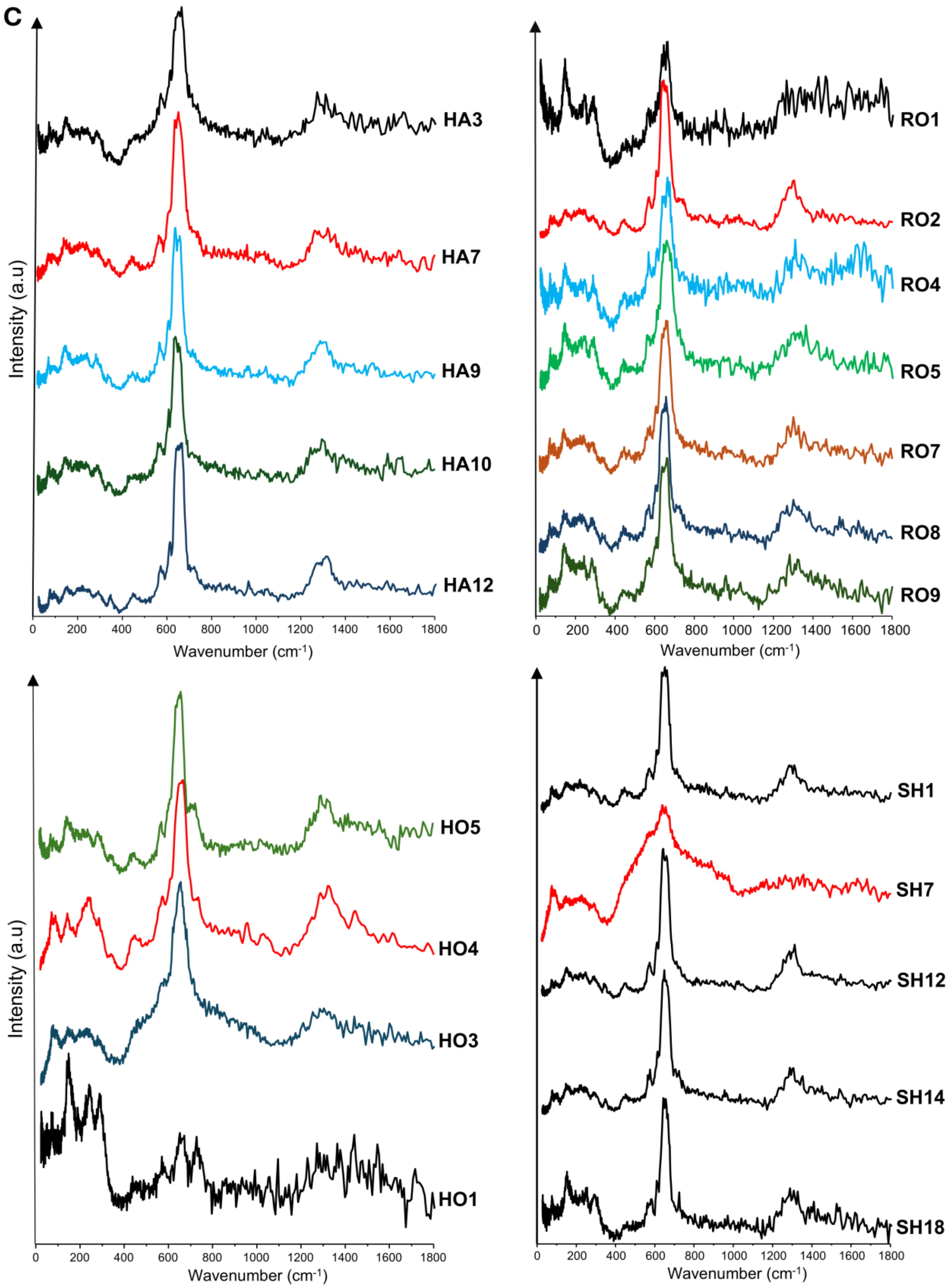

Figure S2. FTIR-ATR (A), Raman (B) and INS (C) spectra of the mummified skeletal samples from Egyptian mummies of the National Museum of Brazil (subjected to fire) analysed in this study (listed in Tab. S1).

References

1. Peters, F.; Schwarz, K.; Epple, M. The structure of bone studied with synchrotron X-ray diffraction, X-ray absorption spectroscopy and thermal analysis. *Thermochimica Acta* **2000,** *361* (1-2), 131.
2. Wang, X. Y.; Zuo, Y.; Huang, D.; Hou, X. D.; Li, Y. B. Comparative study on inorganic composition and crystallographic properties of cortical and cancellous bone. *Biomed Environ Sci* **2010,** *23* (6), 473.
3. Snoeck, C.; Lee-Thorp, J. A.; Schulting, R. J. From bone to ash: Compositional and structural changes in burned modern and archaeological bone. *Palaeogeography Palaeoclimatology Palaeoecology* **2014,** *416*, 55.
4. Wopenka, B.; Pasteris, J. D. A mineralogical perspective on the apatite in bone. *Materials Science & Engineering C-Biomimetic and Supramolecular Systems* **2005,** *25* (2), 131.
5. Festa, G.; Andreani, C.; Baldoni, M.; Cipollari, V.; Martinez-Labarga, C.; Martini, F.; Rickards, O.; Rolfo, M. F.; Sarti, L.; Volante, N.et al. First analysis of ancient burned human skeletal remains probed by neutron and optical vibrational spectroscopy. *Sci Adv* **2019,** *5* (6), eaaw1292.
6. Festa, G.; Rubini, M.; Zaio, P.; Gozzi, A.; Libianchi, N.; Parker, S. F.; Romanelli, G.; de Carvalho, L.; Marques, M. P. M. Vibrational spectroscopy to study ancient Roman funerary practices at the "Hypogeum of the Garlands" (Italy). *Sci Rep* **2022,** *12* (1), 3707.
7. Gonçalves, D.; Rosa, J.; Brandão, A. L.; Martins, A.; Neves, C.; Diniz, M.; Arnaud, J. M.; Marques, M. P. M.; Batista de Carvalho, L. A. E. Infrared Spectroscopy to Assess Manufacturing Procedures of Bone Artefacts from the Chalcolithic Settlement of Vila Nova de São Pedro (Portugal). *Applied Sciences* **2023,** *13* (14).
8. Goncalves, D.; Vassalo, A. R.; Mamede, A. P.; Makhoul, C.; Piga, G.; Cunha, E.; Marques, M. P. M.; Batista de Carvalho, L. A. E. Crystal clear: Vibrational spectroscopy reveals intrabone, intraskeleton, and interskeleton variation in human bones. *Am J Phys Anthropol* **2018,** *166* (2), 296.
9. Mamede, A. P.; Gonçalves, D.; Marques, M. P. M.; Batista de Carvalho, L. A. E. Burned bones tell their own stories: A review of methodological approaches to assess heat-induced diagenesis. *Applied Spectroscopy Reviews* **2017,** *53* (8), 603.
10. Marques, M. P. M.; Goncalves, D.; Mamede, A. P.; Coutinho, T.; Cunha, E.; Kockelmann, W.; Parker, S. F.; Batista de Carvalho, L. A. E. Profiling of human burned bones: oxidising versus reducing conditions. *Sci Rep* **2021,** *11* (1), 1361.
11. Marques, M. P. M.; Mamede, A. P.; Vassalo, A. R.; Makhoul, C.; Cunha, E.; Goncalves, D.; Parker, S. F.; Batista de Carvalho, L. A. E. Heat-induced Bone Diagenesis Probed by Vibrational Spectroscopy. *Sci Rep* **2018,** *8* (1), 15935.
12. Thompson, T. J. U.; Gauthier, M.; Islam, M. The application of a new method of Fourier Transform Infrared Spectroscopy to the analysis of burned bone. *Journal of Archaeological Science* **2009,** *36* (3), 910.
13. Marques, M. P. M.; Batista de Carvalho, L. A. E.; Goncalves, D.; Cunha, E.; Parker, S. F. The impact of moderate heating on human bones: an infrared and neutron spectroscopy study. *R Soc Open Sci* **2021,** *8* (10), 210774.
14. Goncalves, D.; Vassalo, A. R.; Makhoul, C.; Piga, G.; Mamede, A. P.; Parker, S. F.; Ferreira, M. T.; Cunha, E.; Marques, M. P. M.; de Carvalho, L. Chemosteometric regression models of heat exposed human bones to determine their pre-burnt metric dimensions. *Am J Phys Anthropol* **2020,** *173* (4), 734.

15. Hollund, H. I.; Ariese, F.; Fernandes, R.; Jans, M. M. E.; Kars, H. Testing an Alternative High-Throughput Tool for Investigating Bone Diagenesis: Ftir in Attenuated Total Reflection (Atr) Mode*. *Archaeometry* **2012,** *55* (3), 507.
16. Mamede, A. P.; Vassalo, A. R.; Cunha, E.; Goncalves, D.; Parker, S. F.; Batista de Carvalho, L. A. E.; Marques, M. P. M. Biomaterials from human bone - probing organic fraction removal by chemical and enzymatic methods. *RSC Adv* **2018,** *8* (48), 27260.
17. Monnier, G. F. A review of infrared spectroscopy in microarchaeology: Methods, applications, and recent trends. *Journal of Archaeological Science-Reports* **2018,** *18*, 806.
18. Rosa, J.; Vassalo, A. R.; Amarante, A.; Batista de Carvalho, L. A. E.; Marques, M. P. M.; Ferreira, M. T.; Goncalves, D. Burned and buried: A vibrational spectroscopy analysis of burial-related diagenetic changes of heat-altered human bones. *Am J Biol Anthropol* **2023,** *180* (3), 534.
19. Abdel-Maksoud, G.; Ismail, M. Analytical methods for studying mummification technique and degradation process of a human mummy from the late period. *Journal of Cultural Heritage* **2024,** *68*, 237.
20. Cotte, M.; Walter, P.; Tsoucaris, G.; Dumas, P. Studying skin of an Egyptian mummy by infrared microscopy. *Vibrational Spectroscopy* **2005,** *38* (1-2), 159.
21. Edwards, H. G. M.; Farwell, D. W.; Heron, C. P.; Croft, H.; David, A. R. Cats' eyes in a new light: Fourier transform Raman spectroscopic and gas chromatographic mass spectrometric study of Egyptian mummies. *Journal of Raman Spectroscopy* **1999,** *30* (2), 139.
22. Moissidou, D.; Derricott, H.; Kamel, G. Mummified embalmed head skin: SR-FTIR microspectroscopic exploration. *Spectrochim Acta A Mol Biomol Spectrosc* **2021,** *261*, 120073.
23. van Dalen Luna, P.; Majchrzak, L.; Malek, K.; Kuncewicz, J.; Miskowiec, P. The multimodal chemical study of pre-Columbian Peruvian mummies. *Analyst* **2020,** *145* (16), 5670.
24. Bridelli, M. G.; Dell'Anna, A.; Stani, C.; Baraldi, A.; Boano, R.; De Iasio, S. Ft-Ir spectroscopy and microspectroscopy of ancient egyptian embalmed heads from the Museum of Anthropology and Ethnography of the University of Turin. *Journal of Biological Research - Bollettino della Società Italiana di Biologia Sperimentale* **2012,** *85* (1).
25. Martinez Cortizas, A.; Lopez-Costas, O. Linking structural and compositional changes in archaeological human bone collagen: an FTIR-ATR approach. *Sci Rep* **2020,** *10* (1), 17888.
26. Mezzatesta, E.; Dupuy, N.; Mathe, C. Evaluation of a characterization method of Egyptian human mummy balms by chemometric treatments of infrared data. *Talanta* **2021,** *225*, 121949.
27. Petersen, S.; Nielsen, O. F.; Christensen, D. H.; Edwards, H. G. M.; Farwell, D. W.; David, R.; Lambert, P.; Gniadecka, M.; Wulf, H. C. Near-infrared Fourier transform Raman spectroscopy of skin samples from the 'Tomb of the Two Brothers,' Khnum-Nakht and Nekht-Ankh, XIIth dynasty Egyptian mummies (ca 2000 BC). *Journal of Raman Spectroscopy* **2003,** *34* (5), 375.
28. Festa, G.; Romanelli, G.; Senesi, R.; Arcidiacono, L.; Scatigno, C.; Parker, S. F.; Marques, M. P. M.; Andreani, C. Neutrons for Cultural Heritage-Techniques, Sensors, and Detection. *Sensors (Basel)* **2020,** *20* (2).
29. Loong, C. K.; Rey, C.; Kuhn, L. T.; Combes, C.; Wu, Y.; Chen, S.; Glimcher, M. J. Evidence of hydroxyl-ion deficiency in bone apatites: an inelastic neutron-scattering study. *Bone* **2000,** *26* (6), 599.

30. Mamede, A. P.; Vassalo, A. R.; Piga, G.; Cunha, E.; Parker, S. F.; Marques, M. P. M.; Batista de Carvalho, L. A. E.; Goncalves, D. Potential of Bioapatite Hydroxyls for Research on Archeological Burned Bone. *Anal Chem* **2018,** *90* (19), 11556.
31. Taylor, M. G.; Parker, S. F.; Mitchell, P. C. H. A study by high energy transfer inelastic neutron scattering spectroscopy of the mineral fraction of ox femur bone. *Journal of Molecular Structure* **2003,** *651*, 123.
32. Taylor, M. G.; Parker, S. F.; Simkiss, K.; Mitchell, P. C. H. Bone mineral: evidence for hydroxy groups by inelastic neutron scattering. *Physical Chemistry Chemical Physics* **2001,** *3* (8), 1514.
